# Optimization of Apta-Sensing Platform for Detection of Prostate Cancer Marker PCA3

**DOI:** 10.3390/ijms222312701

**Published:** 2021-11-24

**Authors:** Sarra Takita, Alexei Nabok, Anna Lishchuk, David Smith

**Affiliations:** 1 Materials and Engineering Research Institute, Sheffield Hallam University, Sheffield S1 1WB, UK; b5036600@my.shu.ac.uk; 2 Department of Chemistry, The University of Sheffield, Sheffield S3 7HF, UK; a.lishchuk@sheffield.ac.uk; 3 Biomolecular Research Centre, Sheffield Hallam University, Sheffield S1 1WB, UK; hwbds1@exchange.shu.ac.uk

**Keywords:** prostate cancer, PCA3, aptamers, cyclic voltammograms, spectroscopic ellipsometry, XPS

## Abstract

This work is a continuation of our research into the development of simple, reliable, and cost-effective methods for the early diagnosis of prostate cancer (PCa). The proposed method is based on the electrochemical detection of the PCA3 biomarker of PCa (long non-coded RNA transcript expressed in urine) using a specific aptamer labeled with a redox group (methylene blue). The electrochemical measurements (cyclic voltammograms) obtained from electrodes functionalized with the aptamer were complemented in this work by another biosensing technique: total internal reflection ellipsometry (TIRE). In addition to proving the concept of the detection of PCA3 in low concentrations down to 90 pM, this study improved our understanding of the processes by which PCA3 binds to its specific aptamer. The high specificity of the binding of PCA3 to the aptamer was assessed by studying the binding kinetics, which yielded an affinity constant (K_D_) of 2.58 × 10^−9^ M. Additional XPS measurements confirmed the strong covalent binding of aptamers to gold and showed spectral features associated with PCA3 to aptamer binding.

## 1. Introduction

Improving the diagnostic tools used for the early diagnosis of prostate cancer (PCa), one of the most common cancers in men, is of high importance today [1]. The currently used method, which is based on the detection of prostate specific antigen (PSA) in blood serum, is not entirely reliable because of the possibility of both false-positive and false-negative results [2]. Several other biomarkers for prostate cancer are known [3], including PCA3, a long non-coded RNA transcript expressed in the urine of prostate cancer patients, which appears to be an ideal biomarker of PCa [4]. Although PCA3 was discovered a long time ago, until now, its use in prostate cancer diagnosis has been limited to the Progensa PCA assay, licensed in the USA in 2012. This assay detects PCa through PCR amplification of the mRNA of both PCA3 and PSA in the first-catch urine after a digital rectal examination and yields a PCA3 score (the ratio of PCA3 to PSA mRNA molecules in urine specimens) [5,6]. Studies regarding the clinical utility of the PCA3 urine assay for men in Europe and North America confirmed that a PCA3 score cut-off of 35 provides an optimal balance between sensitivity and specificity for PCa detection [6,7]. The diagnostic accuracy is not affected by age, the number of previous negative biopsies, total PSA ranges, or prostate volume [6,7]. Furthermore, this study showed that men with clinical stage T2 or “significant” PCa (biopsy Gleason score ≥ 7) had a substantially higher PCA3 score than men with clinical stage T1 or “indolent” PCa (biopsy Gleason score < 7) [7]. Therefore, the clinical stage and severity of PCa may be related to the PCA3 score. The PCA3 score has recently been connected to tumor volume and Gleason scores in prostatectomy samples [8] and demonstrated its potential in minimizing the number of unnecessary biopsies, as well as the cost of treatment [9].

Despite several advantages, the Progensa test is quite expensive and time-consuming [10,11]. Several attempts have been made recently to develop novel approaches for the detection of PCA3 [12,13,14,15,16], including the use of aptamers as specific bioreceptors for PCA3 [16,17,18]. The use of aptamers is particularly attractive because of their superior stability, simplicity, and low-cost of synthesis which allows for the functionalization of aptamers with various functional groups (i.e., thiols, redox, and fluorescent labels), and introduces the possibility of aptamer recovery in comparison to traditional antibodies [19,20]. The application of aptamers in medical diagnostic tools as bioreceptors for biomarkers of different types of cancers has risen dramatically in the last few years [21,22,23,24,25]. The detection of PCA3 in aptamer assays was reported recently in several publications [17,18,26].

Simple electrochemical methods for the detection of PCA3 using specific aptamers labeled with a redox group (ferrocene) were demonstrated in our previous publication [26]. While the detection principle has been proven, there are still many questions that remain unanswered. For instance, the changes in aptamer configuration when binding to the PCA3 target need further investigation and the concentration of aptamers immobilized on the surface must be optimized to achieve a wider dynamic range of detection. The choice of the aptamer type (DNA or RNA) and the use of different redox labels related to the aptamer type also need to be considered. In this work, we tried to address the above problems using different types of aptamers (RNA-based), including aptamers that are non-labeled and labeled with methylene blue, as well as different experimental methods. In addition to the previously used electrochemical method of cyclic voltammograms, we explored spectroscopic ellipsometry of total internal reflection (TIRE). The processes of aptamer immobilization on the surface of gold and PCA3 to aptamer binding were studied using X-ray photoelectron spectroscopy (XPS).

## 2. Results and Discussion

### 2.1. Electrochemical Detection of PCA3 Using Aptamer Labeled with Methylene Blue

**Figure 1:**
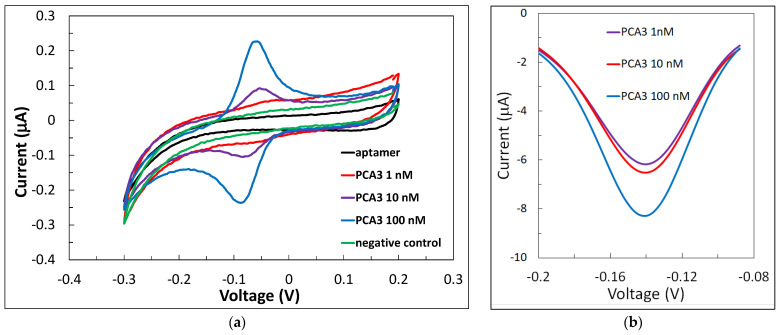
(**a**) A series of CV curves for a 1 μM aptamer layer immobilized on the surface of the SPE before and after binding PCA3 of different concentrations, including a negative control; (**b**) DPV curves for different concentrations of PCA3 bound to the aptamer.

The typical series of cyclic voltammograms (CVs) of gold screen-printed electrodes (SPE) with an immobilized aptamer specific to PCA3 are shown in Figure 1a. The CV of the aptamer layer showed no specific features in the middle of the scan range (−0.3 V to 0.2 V). Binding the target analyte (PCA3) to the aptamer caused the appearance of oxidation and reduction peaks at about −0.05 V and −0.06 to −0.08 V, respectively, which are associated with the oxidation and reduction potentials of methylene blue [27]. The peak intensity increased with an increase in the concentration PCA3. The smallest concentration of PCA3 used was 1 nM, and it yielded barely noticeable peaks. It should be noted that the peak current values increase with an increase in the scan rate, which is expected for systems without redox chemicals in the solution (see Figure S1 in Supplementary Materials). A scan rate of 40 mV/s was chosen as optimal in terms of the signal amplitude and stability. Negative control experiments were carried out using the non-complementary PCA3 molecule with a scrambled sequence of nucleotides. The resulting CV (green curve) has no characteristic features similar to that of freshly deposited aptamers.

Differential pulse voltammetry (DPV) yielded better results (see Figure 1b) with well-resolved reduction peaks at −0.16 V. DPV is the preferable technique for sensing applications. Evaluating the low detection limit was not the goal in this research study, since we aimed to prove the concept of detection; however, the minimum detected concentration of 1 nM is sufficient for PCa diagnosis based on the PCA3 biomarker.

**Figure 2:**
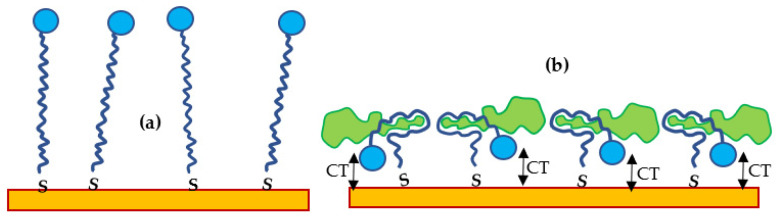
Schematic diagram of redox-labeled aptamers (blue circle) immobilized on the surface of gold before (**a**) and after binding the PCA3 target (green shape), which causes an increase in the charge transfer (CT) (**b**).

The mechanism of detection can be understood from the diagram in Figure 2. In the freshly deposited stretched aptamers in Figure 2a, the methylene blue labels (shown as blue circles) are far away from the gold surface, so that the charge transfer is negligibly small and, therefore, the current peaks do not appear on the CV. The binding process of PCA3 to the aptamer involves the aptamer engulfing the target, thus bringing the redox labels closer to the gold surface and subsequently increasing the charge transfer (CT) and the amplitude of both oxidation and reduction peaks, as illustrated in Figure 2b. Biomarkers non-complementary to the aptamer molecules (PCA3 with a scrambled nucleotide sequence) would not bind to the aptamer and would not cause the above characteristic features in both CV and DPV. Additionally, the non-specific binding of PCA3, which can occur at high concentrations of PCA3 when all the binding sites are occupied and PCA3 molecules pile up on top of the first layer, would not cause an increase in the current since the additional redox labels are away from the surface. It is important to note that the success of DPV and the dependence of redox peaks on the scan rate can be attributed to the limited charge transfer capacity of redox-labeled aptamers.

The charge transfer can be improved by adding redox-active chemicals to the electrolyte; however, this may lead to a completely different concept of detection in which the aptamer layer blocks the charge transfer. Another interesting point to discuss is the concentration of aptamers immobilized on the surface. It seems that increasing the concentration of the aptamers can increase the concentration of binding sites and thus enhance the sensitivity of detection. On the other hand, a high surface concentration of aptamers may not provide sufficient space for aptamer coiling, thus limiting the sensor response. Therefore, the concentration of aptamers should be optimized.

### 2.2. Optical Detection of PCA3 with Non-Labeled Aptamer Using the Method of Total Internal Reflection Ellipsometry (TIRE)

Optical detection of PCA3 with the use of the TIRE method, which does not require the use of labeled aptamers, could provide additional information on the processes associated with PCA3 to aptamer binding. The TIRE method described in detail in Section 3.4 was deployed here for the detection of PCA3 in direct assay with its specific non-labeled aptamers immobilized on the surface of Cr-Au-coated glass slides. The TIRE measurements were performed in standard phosphate buffer solution (PBS; pH 7) on a layer of aptamers before and after binding PCA3 of sequentially increasing concentrations: 0.09, 0.5, 10, and 100 nM. PCA3 solutions of different concentrations (starting from the smallest) were injected into the cell using a syringe and kept there for sufficient time to complete binding. Then, the cell was rinsed with PBS by purging 10× the cell volume. Following that, the steady TIRE spectra were recorded.

**Figure 3:**
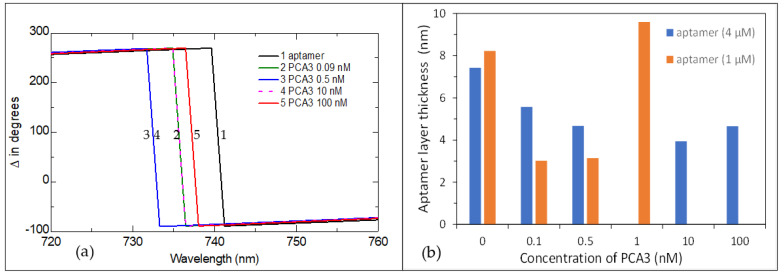
(**a**) A series of TIRE Δ spectra recorded on a 4 μM aptamer layer immobilized on Au (1) and after binding PCA3 in different concentrations: 0.09 (2), 0.5 (3), 10 (4), and 100 nM (5). (**b**) Changes in the thickness of the aptamer layers caused by the binding of PCA3 at different concentrations.

The typical series of the TIRE Δ spectra for the aptamer layer of 4 μM are shown in Figure 3a. The Δ spectra appear to be blue shifted up to a PCA3 concentration of 10 nM, which corresponds to a decrease in the aptamer layer thickness. Then, at the high concentration of PCA3 (100 nM), a red spectral shift occurs, corresponding to an increase in the thickness. The values of thickness of the aptamer layer were found by fitting the TIRE spectra to a four-layer model BK7 glass (prism)/Cr-Au metal layer/aptamer layer/aqueous medium (buffer). The data fitting procedure is explained in detail in our previous publications, for example, [28]. The optical constants of materials: BK7 glass, buffer solution, Cr and Au, were used from the J.A. Woollam library available within VWASE or CompleteEASE software, while the refractive index (*n*) of the aptamer layer is described by the Cauchy model: $n=A_{n}+B_{n}/\lambda^{2}+C_{n}/\lambda^{4},$ with fixed parameters: $A_{n}=1.4,\;B_{n}=0.01,\;\mathrm{and}\;C_{n}=0$, which gives *n* = 1.42, typical for the majority of proteins and polynucleotides including aptamers. The extinction coefficient k = 0 was used since the aptamer layer was optically transparent in the spectral range used [28].

**Figure 4:**
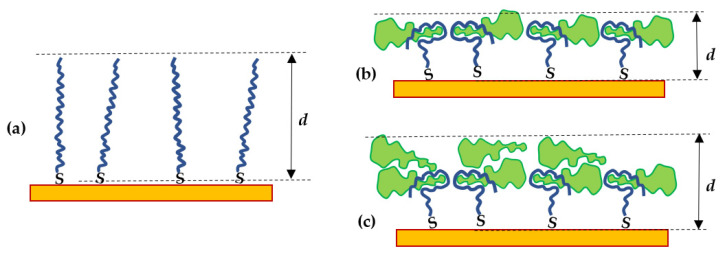
Schematic of a layer (with the thickness *d*) of non-labeled aptamers immobilized on the surface of gold before (**a**) and after binding the PCA3 target (green shapes) accompanied by decreasing *d* (**b**); the case of non-specific adsorption of PCA3 causes an increase in *d* (**c**).

**Figure 5:**
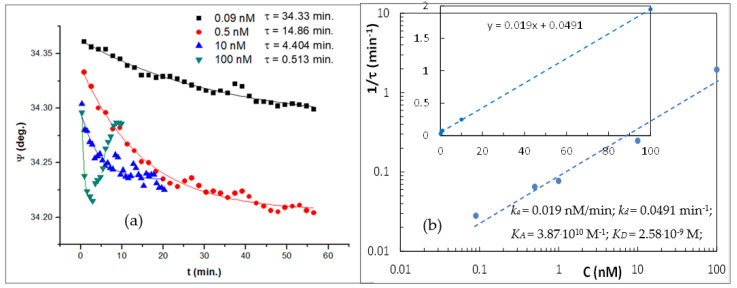
(**a**) Time dependencies of Ψ at 700 nm during the binding of PCA3 at different concentrations to the 4 μM aptamer; (**b**) evaluation of the affinity constant from 1/τ vs. C dependance.

The obtained values of aptamer layer thickness shown in Figure 3b demonstrate the initial decrease in the film thickness at low concentrations of PCA3, followed by an increase in thickness at high concentrations of PCA3. This trend is even more pronounced for low concentrations of aptamers (1 μM). Such behavior can be explained using the model of PCA3 to aptamer binding given in Figure 4. The initial thickness of a layer of freshly immobilized stretched aptamers is typically in the range of 7 to 8 nm (Figure 4a). After binding to PCA3, the thickness of the aptamer layer decreases (as shown in Figure 5b) because the aptamers wrap around the PCA3 target. When all the binding sites (aptamers) are occupied, the binding reaches saturation that can be observed in Figure 4b. The smaller the concentration of the immobilized aptamer, the earlier that saturation occurs. After that, if the concentration of PCA3 increases further, non-specific binding takes place as PCA3 molecules pile on top of the complete layer (see Figure 4c), which increases the aptamer layer thickness, as shown in Figure 4b.

The kinetics of PCA3 to aptamer binding was studied using dynamic TIRE measurements (mentioned in Section 3.4). The typical TIRE kinetics for Ψ fixed at 700 nm was recorded during the binding of PCA3 at different concentrations to aptamers. The typical results are given in Figure 5a. The curves were fitted using exponential decay functions and the obtained values of the time constant (τ) are presented in the graph. The results show that the τ values decrease with an increase in PCA3 concentration. Regarding the TIRE measurements, the selection of the PCA3 incubation time depends on its concentration; the duration of 3τ is sufficient to achieve the saturation of binding.

A noticeable finding is that at the highest concentration of PCA3 (100 nM), the exponential decay in Ψ takes place only in the first 5 min, and it is followed by its increase, which is attributed to the non-specific adsorption of PCA3.

According to the theory of molecular binding to a monolayer of binding sites on the surface (a model suitable for our case of aptamers immobilized on the surface of gold) [29], the reciprocal of the time constant (1/τ) is linearly dependent on the analyte (PCA3) concentration (C): $1/\tau =k_{a}C+k_{d}$, where $k_{a}$ and *k_d_* are the adsorption and desorption rates, respectively. The affinity constant $K_{D}\;\mathrm{can}\;\mathrm{be}\;\mathrm{found}\;\mathrm{as}$ $K_{D}=k_{d}/k_{a}.$ The results in Figure 5a were processed as described above, and the linear dependence of 1/τ vs. PCA3 concentration in Figure 5b yielded the value of the affinity constant: K_D_ = 2.58 × 10^−9^ M. The value obtained is similar to that reported by the founders of the CG-3 RNA aptamer [30], confirming once again the extremely high specificity of this aptamer toward PCA3.

### 2.3. XPX Study

**Figure 6:**
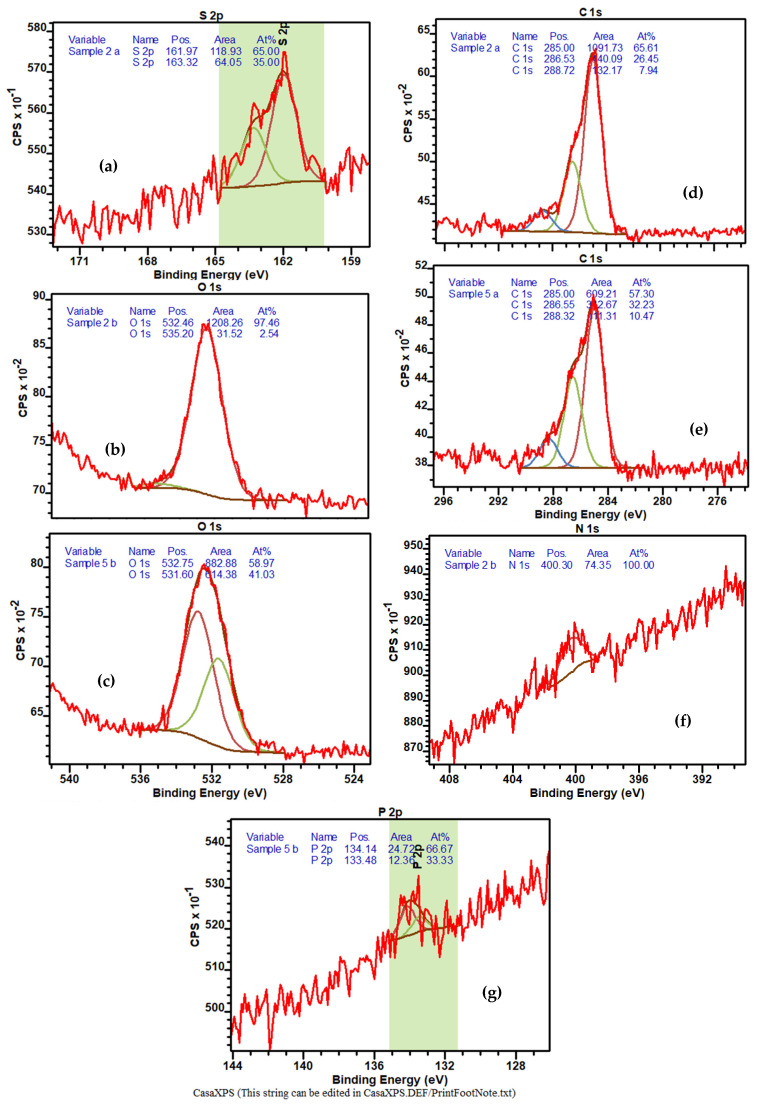
Typical XPS spectral peaks for: S 1 s in aptamer (**a**), O 1 s in aptamer **(b**) and after binding PCA3 (**c**), C 1 s in aptamer (**d**) and after binding PCA3 (**e**), N 1 s (**f**) in aptamer, and P 2p in aptamer after binding PCA3 (**g**).

X-ray photoelectron spectroscopy (XPS) is a common analytical tool for elemental surface analysis, and it has recently been used in the characterization of aptamers immobilized on different surfaces [22,23,24,29]. The samples used for XPS measurements were prepared by immobilizing aptamers (from their 1 µM and 4 µM solutions) on Cr-Au-coated glass slides followed by drop-casting PCA3 solutions of two different concentrations (low, 0.01 nM and high, 10 nM). Typical XPS spectra are summarized in Figure 6.

To confirm the successful immobilization of aptamers on the surface of Au, the peaks of S 2p were analyzed. All samples containing immobilized aptamers showed two characteristic peaks at about 162 and 163.3 eV (Figure 6a), which correspond to the S of the aptamers’ thiol groups bound to Au [29], while there were no such peaks on the bare Au surface. The characteristic XPS peaks associated with O 1s are shown in Figure 6b,c. It should be noted that in addition to a single O 1s peak at about 532.5 eV (Figure 6b), associated with C–O bonding [29] in the aptamer layer, a second peak appeared at about 532.5 eV after binding PCA3 (Figure 6c). The latter peak associated with H–C–O bonding [31] indicates the formation of a hydrogen bond between the aptamer and PCA3. XPS spectra for C 1s in Figure 6d and e show three peaks at about 285, 286.5, and 288.5 eV corresponding to the C–C, C–O, and O–C=O bonds, respectively [31]. The increase in the C–O and O–C=O peaks in Figure 6e can be attributed to the formation of hydrogen bonds between the aptamer and PCA3 upon bending. The XPS peak related to N 1s at about 400.3 eV in Figure 6f, which appeared only on samples with aptamers, is quite small, and its intensity slightly increases after binding PCA3. Finally, the XPS peaks of P 2p at about 134.1 and 133.5 eV in Figure 6g are quite small and appeared on samples with aptamers after binding PCA3. Changes in the concentration of both immobilized aptamers and aptamers bound to PCA3 did not have significant effects on the XPS spectra and, in some samples, the peak intensities were smaller for lower concentrations. All the results shown in Figure 6b–g confirmed the formation of the aptamer-PCA3 complex on the surface.

## 3. Materials and Methods

### 3.1. Aptamers and Target Analyte

The HPLC-purified CG-3 RNA-based aptamer, 5′-MHB-AGUUUUUGCGUGUGCCCUUUUUGUCCCC-SH-3′ specific to 277 bases of PCA3 transcript, was purchased from Merck Life Science UK Limited, (Dorset, England). This CG-3 RNA-based aptamer sequence generated against the 277 nt section of the PCA3 transcript by SELEX technology was first reported by Marangoni et al. [30]. The aptamers were functionalized with a thiol (SH) group at C3′ termini for further immobilization on the surface of gold. The redox functional groups of methylene blue attached at the C5′ termini were used as electron mediators to provide distinctive electrochemical properties. A batch of aptamers without redox labels was also produced. Aptamers delivered in the lyophilized form were then dissolved in Tris buffer (10 mM TRIS-HCL, 0.5 mM disodium EDTA, pH 7.8) and stored at −20 °C in small aliquots to avoid repeating thaw-freeze cycles.

The target analyte, a 78 bp fragment of lncRNA-PCA3 having a molecular mass of 25,048.3 g/mol, was purchased from Eurofins Scientific (Manchester, UK) and prepared in PBS (pH 7.5). All other chemicals used were purchased from Sigma-Aldrich (Haverhill UK) which included the HEPES and phosphate binding buffers (HBB and PBB) and 1,4-dithiothreitol (DTT). All aqueous solutions were prepared using 18.2 MX cm ultra-pure water (Millipore Ltd., Livingston, UK) with a pyro Gard filter (Millipore) to remove nucleases.

### 3.2. Immobilization of Aptamers, Electrochemical and Optical Sensor Fabrication

The aptamer stock solution was diluted to desired concentrations of 1 and 4 μM in the immobilization buffer, which consisted of HEPES binding buffer (HBB) (pH 7.4), supplemented with 1 mM of 1,4-dithiothreitol (DTT), and 3 mM of MgCl_2_. The addition of MgCl_2_ was essential to preserve the aptamer single RNA strand from self-coiling. Unreacted aptamers were removed from the electrode surface by several rinses with a non-folding buffer. Before immobilization, the aptamer liquid samples were activated by rapid heating to 95 °C for 5 min, followed by 5 min of cooling at 4 °C in a thermocycler (Prime TC3600) to achieve the correctly unfolded structure.

For electrochemical measurements, the redox-labeled aptamers were immobilized on the surface of gold screen-printed three-electrode assemblies (DropSens, Metrohm UK Ltd., Runcorn, UK) cleaned by sonication in acetone for 3 min and dried with nitrogen gas flow. Immobilization of aptamers was carried out by casting an aptamer solution (about 40 μL onto the surface of the screen-printed gold electrodes) and the samples were then incubated for 5 h at room temperature in a humidity chamber. The same immobilization protocol was used for the non-labeled aptamers during sample preparation for optical spectroscopic ellipsometry measurements. The samples were based on BK7 microscopic glass slides cleaned in Piranha solution, rinsed with de-ionized water, and dried under nitrogen gas flow. The layers of Cr (3–5 nm) and Au (20–25 nm) were deposited on clean glass slides using thermal evaporation in an Edwards 360 evaporation unit under a vacuum of 10^−6^ Torr. A thin layer of Cr was used to improve the adhesion of the Au layer to the glass. Both the screen-printed gold electrodes and the Cr-Au-coated glass slides with immobilized aptamers were kept at 4 °C in HBB to prevent the aptamers from coiling.

The lncRNA-PCA3 target analytes were re-suspended in electrolyte buffer (HEPES pH 7.5, 120 mm NaCl, and 5 mm KCl) at different concentrations from 100 nM down to 0.09 nM and were used in electrochemical measurements. PCA3 solutions in HEPES buffer (pH 7.5) of similar concentrations were used in optical spectroscopic ellipsometry measurements.

### 3.3. Cyclic Voltammogram Measurements

Cyclic voltammogram (CV) measurements were carried out on three-electrode DropSens gold screen-printed assemblies with Ag/AgCl as the reference electrode using potentiostat STAT8000P from DropSens (Metrohm UK Ltd., Runcorn, UK). A voltage range from −0.3 to 0.2 V was used in CV measurements with an optimized scan rate (40 mV/S) recorded 5 times until the current readings were stabilized. In addition to cyclic voltammetry, the differential pulse voltammetry (DPV) of PCA3 under different concentrations was recorded using a pulse amplitude of 20 mV and a scan rate of 10 mV/s. Both CV and DPV measurements were carried out on electrodes with immobilized aptamers immersed in an electrolyte buffer containing different concentrations of PCA3 from 1 ng/mL to 1000 ng/mL; measurements of buffer alone were used as a reference. Negative control measurements were carried out using non-complementary fragments of lncRNA-PCA3 with a scrambled *nt* sequence.

### 3.4. Spectroscopic Ellipsometry Measurements

**Figure 7:**
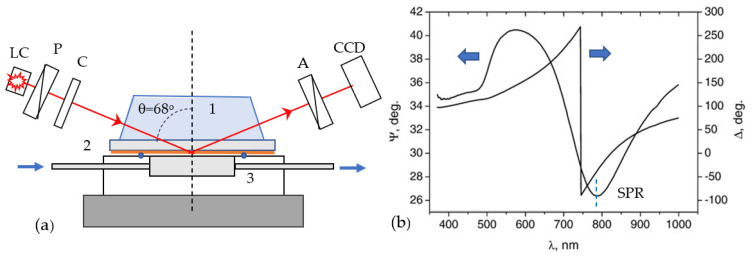
(**a**) TIRE experimental set-up based on the spectroscopic ellipsometer instrument with a light source (LC), polarizer (P), compensator (C), analyzer (A), and a CCD photodetector with the SPR unit attachment consisting of a glass prism (1), a Au-coated glass slide (2), and a PTFE cell (3) placed on the ellipsometer stage; (**b**) typical TIRE spectra of Ψ and Δ recorded on a Cr-Au-coated glass slide with a layer of aptamers immobilized on the Au surface.

The method of total internal reflection ellipsometry (TIRE) was used in this work as an alternative experimental technique for the quantification of PCA3 to aptamer binding. TIRE, also known as phase SPR (surface plasmon resonance), was experimentally realized by Poksinsky and Arvin [32] in 2005 as a combination of spectroscopic ellipsometry instrumentation and SPR total internal reflection geometry. This method was quickly adapted by our research group and successfully applied for different biosensing applications [33,34,35,36,37]. The experimental set-up shown in Figure 7a is based on the spectroscopic ellipsometer M2000 (J. A. Woollam Co., Lincoln, NE, USA) with the addition of a 68° glass prism, which provides total internal conditions for light coupling into glass slides coated with a thin Cr-Au layer. A PTFE cell of 0.2 mL in volume was sealed to the gold side of a slide. The whole assembly of the prism/gold-coated slide/cell was placed on the ellipsometer stage. In contrast to traditional SPR equipment recording the intensity of reflected light, in the TIRE method, the spectra of two parameters Ψ and Δ are recorded, where Ψ is the ratio of amplitudes of the *p*- (in the plane of incidence) and *s*- (normal to the plane of incidence) components of the electric field vector of polarized light ($tan\left(\Psi \right)=E_{p}/E_{s}$), and Δ is the phase shift between $E_{p}$ and $E_{p}$ ($\Delta =\phi_{p}-\phi_{s}$).

The typical TIRE spectra of Ψ and Δ are shown for Cr-Au-coated slides with the layer of bioreceptors (aptamers) in contact with the aqueous medium (buffer) in Figure 7b. The spectrum exhibits an SPR-like shape for Ψ, with the minimum corresponding to plasmon resonance, while the Δ spectrum shows a sharp drop near the resonance position at which it appears to be extremely sensitive to changes in the thickness or refractive index of the molecular layer [35]; that is why the TIRE method is particularly beneficial for the detection of small molecules such as mycotoxins [33,34,35,36,37]. Typically, an increase in the layer thickness corresponds to a red TIRE spectral shift. The TIRE method also allowed for studying the kinetics of molecular adsorption (or binding) by recording the Ψ and Δ spectra many times after a certain time interval during the process of molecular adsorption and then plotting the values of Ψ or Δ at a particular wavelength vs. time. Both steady-state and dynamic TIRE measurements were carried out in this work.

### 3.5. X-ray Photoelectron Spectroscopy (XPS) Measurements

XPS measurements were performed using a Kratos Supra X-ray photoelectron spectrometer (Kratos Analytical, Manchester, UK) with a monochromatic Al Kα X-ray source (1486 eV). The area of analysis was 300 × 700 µm. The survey spectra were acquired at pass energy of 160 eV. High-resolution scans for C 1s, N 1s, O 1s, Au 4f, S 2p, and P 2p spectral bands were collected over appropriate energy ranges. All XPS spectra were analyzed and curve-fitted using the Casa XPS software; all spectra were calibrated relative to the C 1 s signal at a binding energy of 285.0 eV. Measurements were repeated twice, and the errors quoted in the numerical data represent the standard deviations.

## 4. Conclusions

The study of binding a fragment of the 277 nt lncRNA transcript of PCA3 to a specific CG-3 RNA aptamer was carried out using several experimental methods: cyclic voltammograms, total internal reflection ellipsometry, and X-ray photoelectron spectroscopy. CV and DPV measurements proved the concept of electrochemical detection of the prostate cancer marker PCA3 using aptamers labeled with methylene blue. This method appeared not to be affected by non-specific adsorption of PCA3 at high concentrations. This work was not focused on the evaluation of the sensitivity of both the electrochemical and optical detection, but rather on the qualitative comparison of the two methods. The smallest concentration of PCA3 used in this work was 1 nM, which is equivalent to 2.5 ng/mL (considering the molecular weight of about 2.5 kD for a 78 bp fragment of lncRNA-PCA3 transcript). Our previous study based on the same strategy of electrochemical detection of PCA3 using a redox-labeled aptamer [26] demonstrated a wide detection range of PCA3 from 1 μg/mL down to 0.1 ng/mL. The sensitivity of the proposed electrochemical aptasensor is comparable to the values reported in [11,12,13] and is thought to be sufficient for the detection of the PCA3 prostate cancer biomarker in urine.

The concentration of immobilized aptamers must be optimized to achieve the highest sensitivity. Optical TIRE measurements that show higher sensitivity than CV can distinguish between the specific and non-specific binding of PCA3 and can confirm the model of aptamer/PCA3 interaction. The XPS measurements provide evidence of strong covalent binding of aptamers to gold via thiol groups, as well as the interaction between PCA3 and the aptamer. This study provided us with a better understanding of processes associated with the aptamer/PCA3 interaction, which is a significant step toward the development of a simple, cost-effective, and reliable methodology for the early diagnosis of prostate cancer. Further work should be directed toward optimizing the concentration of immobilized aptamers to achieve the highest sensitivity of PCA3 detection. Future work will focus on the use of methods such as DPV, impedance spectroscopy (IS), and TIRE, which appear to be more sensitive than CV. Additionally, the detection of both PCA3 and PSA in a complex media of urine is required for further development of quantitative PCa diagnostic tools.

## Data Availability

Not applicable.

## Supplementary Materials

The following are available online at https://www.mdpi.com/article/10.3390/ijms222312701/s1.
