# Supplementary material for: Optimization of Apta-Sensing Platform for Detection of Prostate Cancer Marker PCA3
Source: Int J Mol Sci. 2021 Nov 24;22(23):12701. doi: 10.3390/ijms222312701 (PMC8657731; doi:10.3390/ijms222312701)
Supplement: Supplementary file 1 [file ijms-22-12701-s001.zip › ijms-1466909-supplementary.pdf]

Supplementary Materials

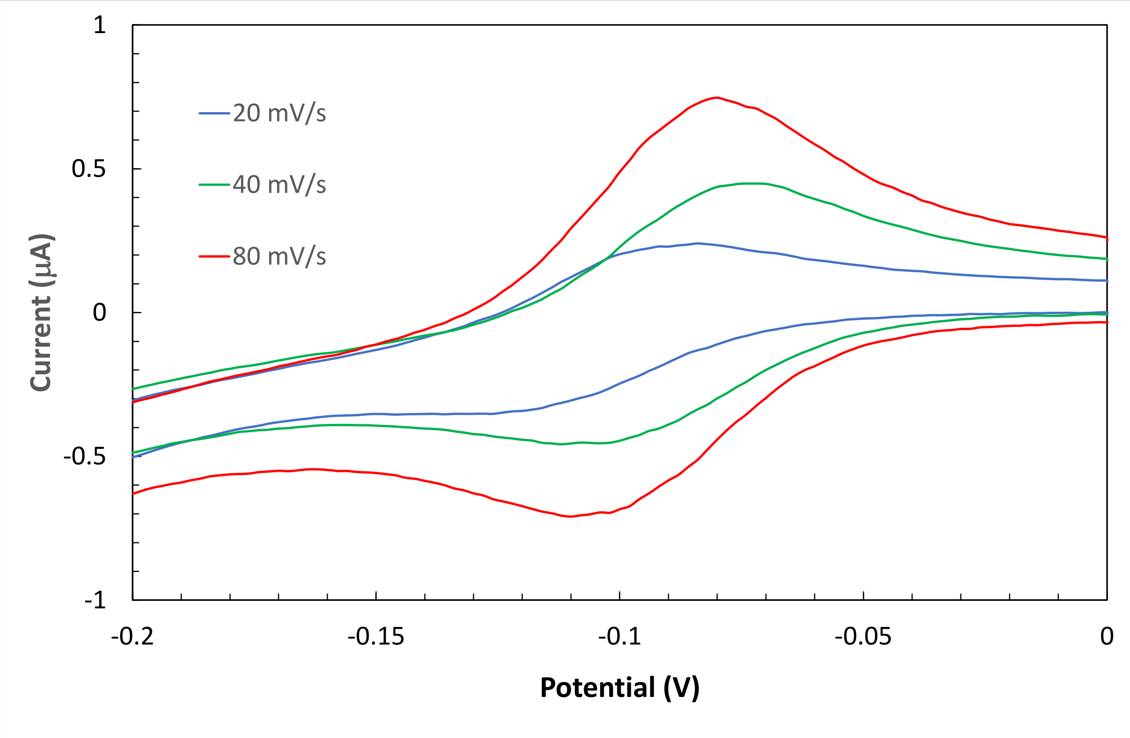

Figure S1. CV graphs recorded at different scan rates on electrodes with immobilized aptamer after binding PCA3 from its 100 nM solution.

These data showed that the current values are directly proportional to the scan rate which is a characteristic for charge transfer between the redox label and the electrode. The observed shifts in the peak positions (particularly for the oxidation peak) could be due to instability of CV at high scan rates, which means that more scans are required to achieve stable CV curve. The scan rate of 40 mV/s appeared to be optimal in terms of the current amplitude and stability of CV.
